# Design and baseline characteristics of the ILD-PRO registry in patients with progressive pulmonary fibrosis

**DOI:** 10.1186/s12890-024-03247-8

**Published:** 2024-09-27

**Authors:** L Jason Lobo, Yi Liu, Peide Li, Murali Ramaswamy, Aparna C Swaminathan, Srihari Veeraraghavan, Yanni Fan, Megan L Neely, Scott M Palmer, Amy L Olson, on behalf of the ILD-PRO Registry investigators†

**Affiliations:** 1 UNC School of Medicine, Chapel Hill, NC USA; 2 Boehringer Ingelheim Pharmaceuticals, Inc, Ingelheim, Germany; 3 Boehringer Ingelheim Pharmaceuticals, Inc, Ridgefield, CT USA; 4 LeBauer Pulmonary and PulmonIx LLC at Cone Health, Greensboro, NC USA; 5 Duke Clinical Research Institute, Durham, NC USA; 6 Duke University Medical Center, Durham, NC USA; 7 Emory Healthcare, Atlanta, GA USA; 8 101 Manning Dr, Chapel Hill, NC 27514 USA

**Keywords:** Clinical trial, Disease progression, Interstitial lung disease, Pulmonary fibrosis

## Abstract

**Background:**

To assess the characteristics of patients enrolled in the ILD-PRO Registry.

**Methods:**

The ILD-PRO Registry is a multicentre US registry of patients with progressive pulmonary fibrosis. This registry is enrolling patients with an interstitial lung disease (ILD) other than idiopathic pulmonary fibrosis who have reticular abnormality and traction bronchiectasis on HRCT, and who meet criteria for ILD progression within the prior 24 months. Patient characteristics were analysed based on the number of patients with available data.

**Results:**

Of the first 491 patients enrolled, the majority were white (75.4%) and female (60.6%); 47.4% had a history of smoking. Reported ILDs were autoimmune disease-associated ILDs (47.2%), hypersensitivity pneumonitis (17.5%), idiopathic non-specific interstitial pneumonia (9.1%), interstitial pneumonia with autoimmune features (8.9%), unclassifiable ILD (7.6%), other ILDs (9.7%). At enrolment, median (Q1, Q3) FVC % predicted was 62.2 (49.4, 72.4) and DLco % predicted was 39.2 (30.2, 49.2). Median (Q1, Q3) total score on the St. George’s Respiratory Questionnaire was 50.8 (35.9, 64.7). The most common comorbidities were gastroesophageal reflux disease (61.1%) and sleep apnoea (29.6%). Overall, 64.5% of patients were receiving immunosuppressive or cytotoxic therapy, 61.1% proton-pump inhibitors, 53.2% oral steroids, 19.8% nintedanib and 3.6% pirfenidone.

**Conclusions:**

Patients enrolled into the ILD-PRO Registry have a variety of ILD diagnoses, marked impairment in lung function and health-related quality of life, and high medication use. Longitudinal data from this registry will further our knowledge of the course of progressive pulmonary fibrosis.

**Trial Registration:**

ClinicalTrials.gov, NCT01915511; registered August 5, 2013.

**Supplementary Information:**

The online version contains supplementary material available at 10.1186/s12890-024-03247-8.

## Introduction

Interstitial lung diseases (ILDs) comprise a large and heterogeneous group of parenchymal lung disorders [1]. ILD may result in pulmonary fibrosis. Idiopathic pulmonary fibrosis (IPF) is a type of ILD that is always associated with progressive pulmonary fibrosis [2]. However, the term “progressive pulmonary fibrosis” or “PPF” is generally used to refer to progressive pulmonary fibrosis in patients with ILDs other than IPF [2, 3]. Various criteria have been proposed to identify patients with PPF, based on decline in lung function, worsening radiologic abnormalities, and worsening symptoms [2, 4–9]. Patients identified based on these criteria have poor outcomes, including high mortality [8, 9].

The IPF-PRO Registry (NCT01915511) enrolled 1002 patients with IPF at 46 centres across the US [10]. In 2018, the IPF-PRO Registry was expanded to include patients with progressive fibrosing ILDs other than IPF to form the IPF-PRO/ILD-PRO Registry. This registry is supported by Boehringer Ingelheim Pharmaceuticals, Inc (BIPI) and run in collaboration with the Duke Clinical Research Institute (DCRI) and enrolling centres. The ILD-PRO Registry aims to explore the clinical course of PPF, including clinical, radiologic and blood-based predictors of progression, its impact on patients, and treatment practices. Here, we describe the design of the ILD-PRO Registry and the demographic and clinical characteristics of the first group of patients enrolled.

## Study design and methods

### Patients

The ILD-PRO Registry is enrolling patients aged ≥ 30 years with a non-IPF ILD of any duration that was diagnosed or confirmed at the enrolling centre, with reticular abnormality and traction bronchiectasis (with or without honeycombing) confirmed by HRCT scan and/or lung biopsy. Patients must meet ≥ 1 of the following criteria for ILD progression at any time within the past 24 months: relative decline in FVC % predicted ≥ 10%; relative decline in DLco % predicted ≥ 10%; relative decline in FVC % predicted ≥ 5–<10% plus worsened respiratory symptoms; relative decline in FVC % predicted ≥ 5–<10% plus increased extent of fibrotic changes on HRCT; worsened respiratory symptoms plus increased extent of fibrotic changes on HRCT. In the case report form, investigators are asked to select a single criterion that best applies to the subject.

Patients with malignancy (other than skin or early-stage prostate cancer) within the prior 5 years are not eligible to participate. Patients listed for lung transplantation or enrolled in a clinical trial are not eligible for enrolment, but patients may be listed for lung transplant or enter a clinical trial after enrolment.

The study was approved by the Duke University Institutional Review Board (Pro00046131). The protocol was also approved by the relevant Institutional Review Boards and/or local Independent Ethics Committees prior to patient enrolment at each site listed in the Supplementary Appendix (Additional file 2). All patients are required to provide written informed consent.

## Data collection

**Fig. 1:**
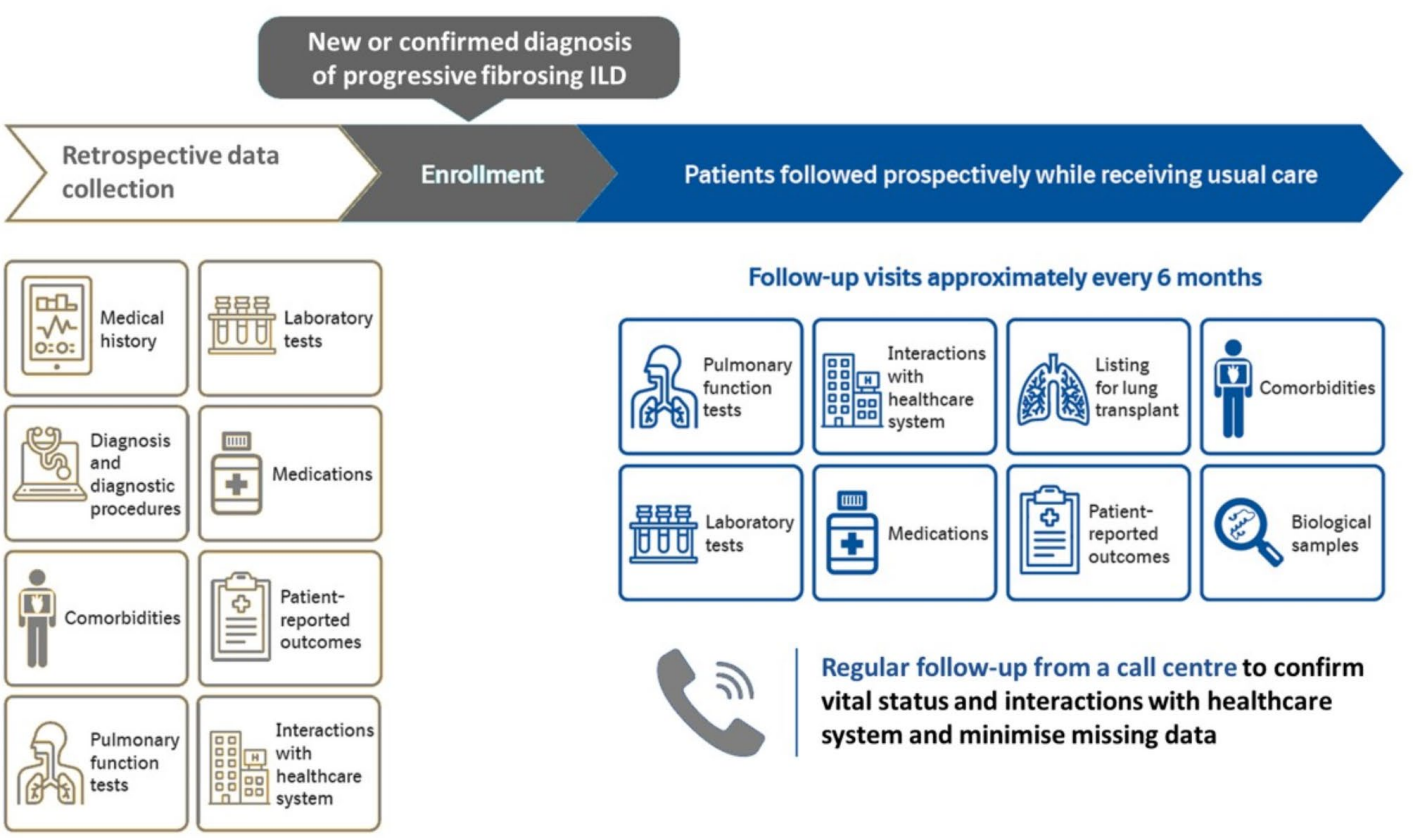
Design of ILD-PRO Registry

At enrolment, the diagnosis provided by the site investigator is recorded. Retrospective data from the prior 24 months are collected from medical records, including data on medical history, comorbidities, pulmonary function tests and medications. Patients are then followed prospectively while receiving usual care. Regular follow-up from a call centre enables confirmation of vital status and interactions with healthcare systems (Fig. 1).

Patient-reported outcomes are assessed at enrolment and subsequent clinic visits. The St. George’s Respiratory Questionnaire (SGRQ) comprises domains measuring the frequency and severity of symptoms, the effects of dyspnoea on activities, and the psychological/social impact of the disease [11]. The Cough and Sputum Assessment Questionnaire (CASA-Q) includes domains assessing cough symptoms and their impact [12]. The 12-item short-form survey (SF-12) includes components to assess mental and physical health [13]. The EuroQoL index score and visual analogue scale (VAS) assess overall health status [14].

HRCT scans obtained within the 24 months prior to enrolment and during follow-up will be used for qualitative and quantitative assessments. Blood samples will be collected at enrolment and subsequent clinic visits to provide biological samples for investigation of biomarkers. Whole blood samples for DNA and RNA analysis, as well as isolated plasma and serum samples, will be collected.

### Analyses

Data for these analyses were abstracted from the database in September 2022. Patient characteristics at enrolment were analysed in all patients and in subgroups by the following types of ILD: autoimmune disease-associated ILDs, hypersensitivity pneumonitis (HP), idiopathic non-specific interstitial pneumonia (iNSIP), interstitial pneumonia with autoimmune features (IPAF), unclassifiable ILD, and other ILDs. Patient characteristics at enrolment were also assessed in subgroups based on a shorter versus longer (below versus above the median) time from ILD diagnosis to enrolment. The characteristics of patients in the ILD-PRO Registry were compared with the characteristics of patients in the IPF-PRO Registry.

Continuous variables are presented as median (Q1, Q3) and categorical variables as the number and percentage of patients with available data. P-values for comparisons between patients with a shorter versus longer time from ILD diagnosis to enrolment in the ILD-PRO Registry, and between patients enrolled in the IPF-PRO and ILD-PRO registries, were based on two-sample t-tests (for continuous variables) or two-proportion tests or Chi-square tests (for categorical variables).

## Results

### Patient characteristics at enrolment in the ILD-PRO Registry

**Fig. 2:**
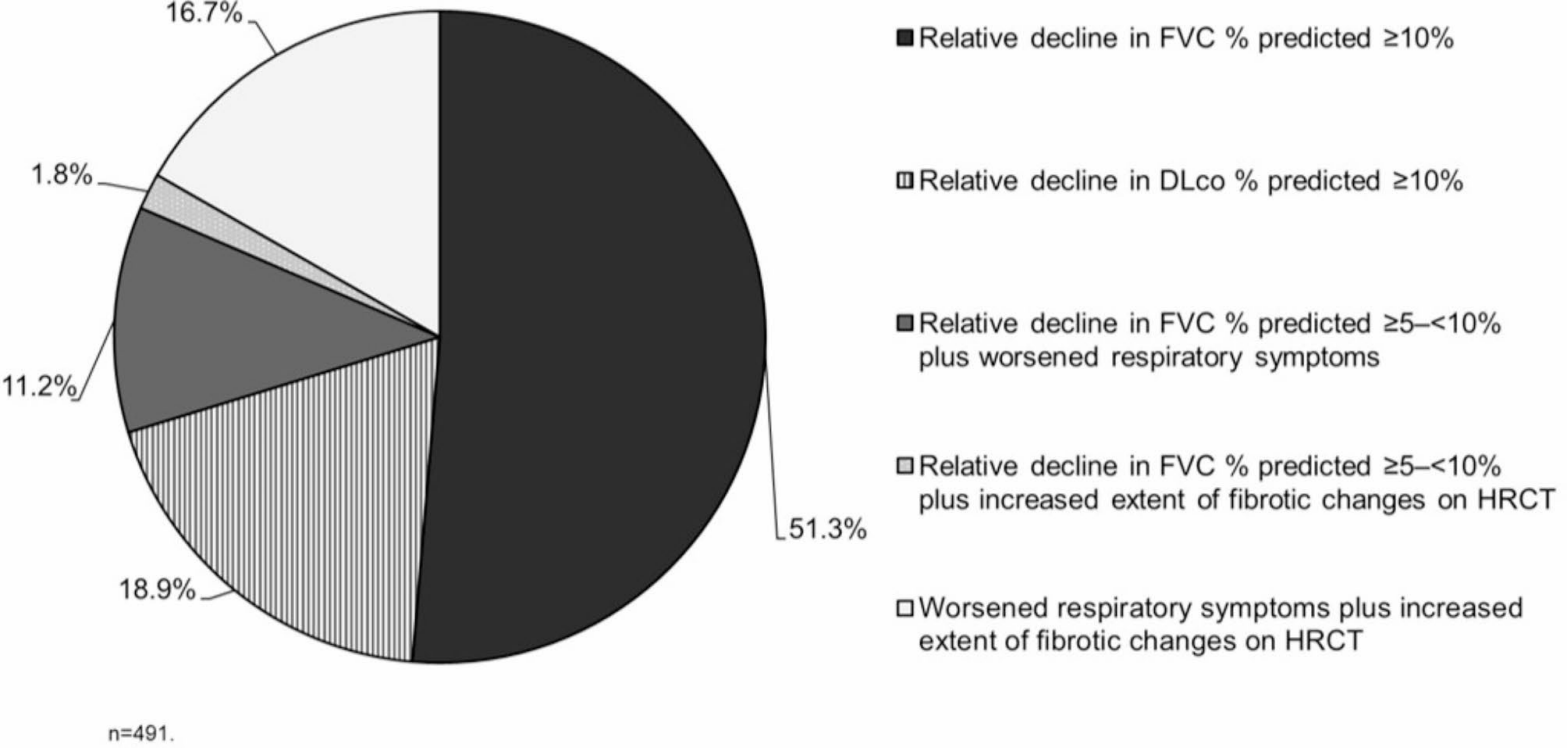
Inclusion criteria related to ILD progression in patients in the ILD-PRO Registry. Investigators were asked to select the criterion that best applied to the subject

**Fig. 3:**
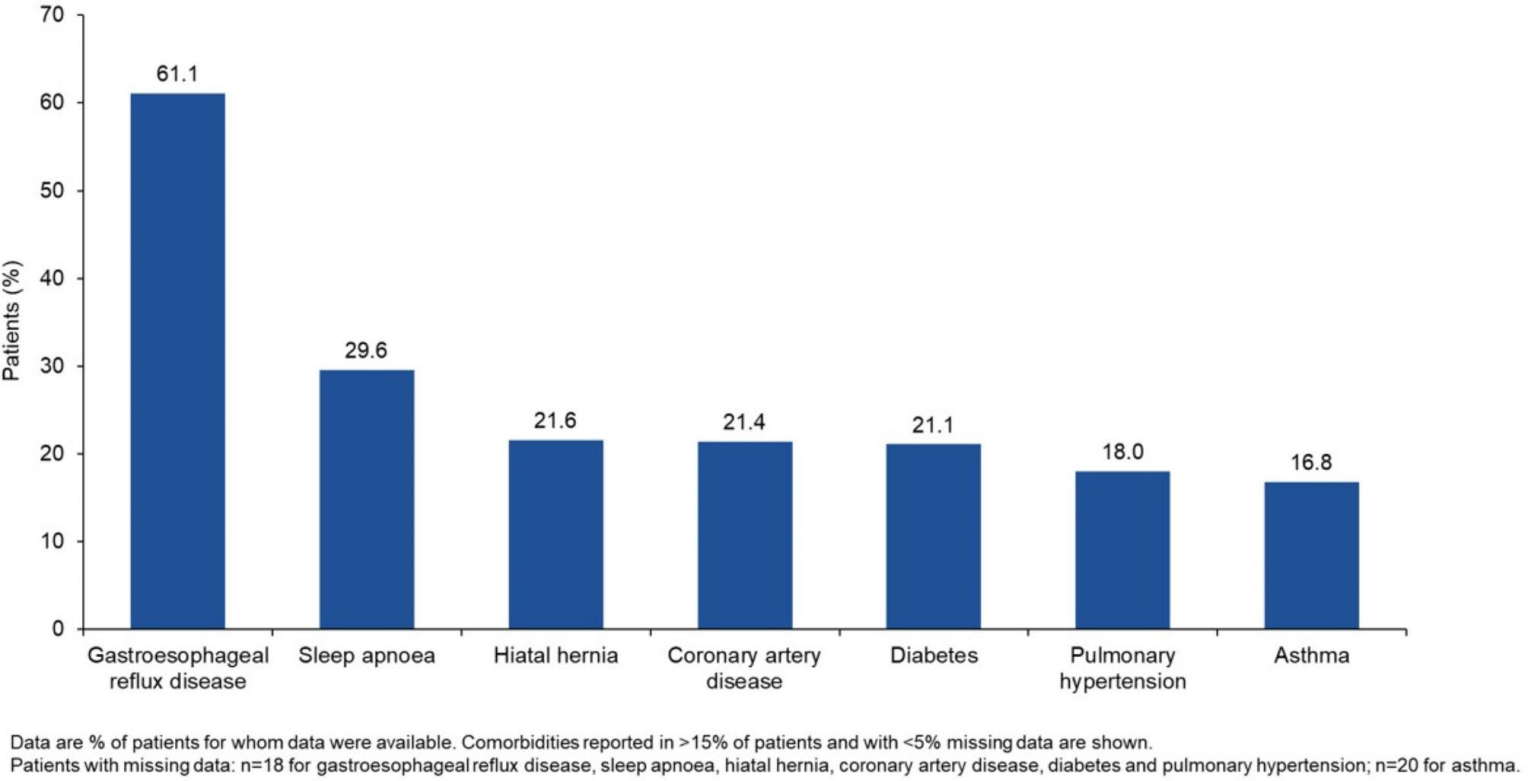
Comorbidities at enrolment into the ILD-PRO Registry

**Fig. 4:**
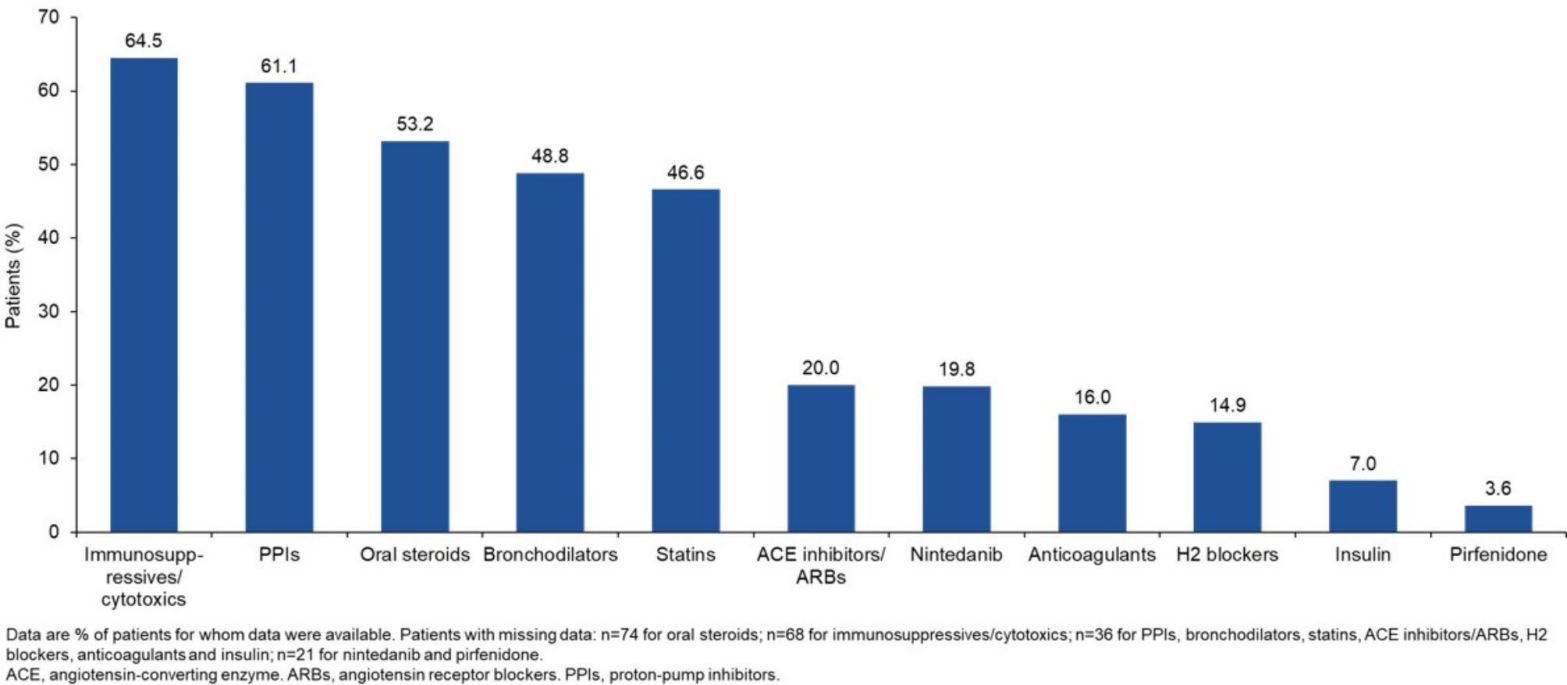
Medications used at enrolment into the ILD-PRO Registry

This analysis used data from the first 491 patients enrolled in the ILD-PRO Registry. The majority of patients were white (75.4%) and female (60.6%); 47.4% of patients had a history of smoking (Table 1). Median (Q1, Q3) FVC % predicted was 62.2 (49.4, 72.4) and DLco % predicted was 39.2 (30.2, 49.2). Patient-reported outcomes indicated impairment in health-related quality of life (Table 2). For approximately half (51.3%) of the patients enrolled in the registry, the investigator selected a relative decline in FVC % predicted ≥ 10% within the prior 24 months as the criterion related to ILD progression that best applied to that patient (Fig. 2). The most common comorbidities reported at enrolment were gastroesophageal reflux disease (61.1%) and sleep apnoea (29.6%) (Fig. 3). Overall, 64.5% of patients were receiving immunosuppressive or cytotoxic therapy, 61.1% proton-pump inhibitors, 53.2% oral steroids, 19.8% nintedanib and 3.6% pirfenidone (Fig. 4). A summary of the immunosuppressive or cytotoxic therapies received is provided in Additional file 3.

**Table 1:** Demographic and clinical characteristics at enrolment into the ILD-PRO Registry

	Autoimmune disease-associated ILDs (*n* = 229)	HP (*n* = 85)	iNSIP (*n* = 44)	IPAF (*n* = 43)	Unclassifiable ILDs (*n* = 37)	Other ILDs (*n* = 47)	Total (*n* = 491)
Female	154 (67.5)	53 (62.4)	27 (61.4)	25 (58.1)	12 (32.4)	21 (44.7)	297 (60.6)
Age, years	66.0 (55.0, 72.0)	69.0 (64.0, 74.0)	65.5 (60.0, 74.5)	70.0 (58.0, 77.0)	71.0 (64.0, 75.0)	66.0 (50.0, 72.0)	67.0 (58.0, 74.0)
Race							
White	147 (66.2)	77 (95.1)	29 (69.0)	34 (81.0)	32 (86.5)	36 (76.6)	359 (75.4)
Black/African-American	59 (26.6)	1 (1.2)	8 (19.0)	7 (16.7)	3 (8.1)	10 (21.3)	89 (18.7)
Other	16 (7.2)	3 (3.7)	5 (11.9)	1 (2.4)	2 (5.4)	1 (2.1)	28 (5.9)
Current or former smoker	88 (39.8)	41 (51.3)	23 (52.3)	20 (50.0)	20 (54.1)	29 (61.7)	224 (47.4)
Time from ILD diagnosis to enrolment, years	2.2 (1.1, 4.3)	2.0 (1.0, 4.6)	1.6 (1.0, 4.3)	2.1 (0.9, 3.4)	1.2 (0.8, 2.5)	2.0 (1.3, 4.1)	2.0 (1.0, 4.1)
Family history of ILD	19 (8.7)	7 (8.9)	11 (25.0)	3 (7.7)	5 (13.9)	5 (10.6)	52 (11.1)
Hospitalization within prior 12 months	57 (26.1)	22 (27.8)	13 (29.5)	18 (46.2)	8 (22.2)	14 (29.8)	133 (28.4)
FVC, L	2.0 (1.5, 2.6)	1.9 (1.4, 2.4)	2.2 (1.6, 2.8)	2.0 (1.6, 2.7)	2.5 (2.1, 3.2)	2.3 (1.8, 3.0)	2.0 (1.6, 2.7)
FVC, % predicted	61.8 (49.5, 70.5)	56.7 (45.1, 65.2)	67.9 (52.0, 77.8)	62.4 (47.0, 77.5)	67.5 (52.9, 79.6)	68.6 (54.6, 77.4)	62.2 (49.4, 72.4)
FEV_1_, L	1.6 (1.3, 2.0)	1.5 (1.2, 2.0)	1.7 (1.3, 2.3)	1.7 (1.3, 2.2)	2.1 (1.6, 2.8)	1.9 (1.5, 2.4)	1.7 (1.3, 2.1)
DLco, mL/min/mmHg	9.8 (7.1, 12.9)	10.1 (8.9, 12.5)	10.5 (7.6, 14.1)	10.8 (7.9, 14.7)	12.1 (9.4, 15.0)	11.7 (9.5, 14.9)	10.3 (7.8, 13.4)
DLco, % predicted	37.1 (28.9, 46.3)	40.3 (31.8, 48.0)	39.2 (30.6, 52.1)	38.0 (30.0, 50.1)	42.8 (31.4, 49.6)	39.4 (30.4, 52.1)	39.2 (30.2, 49.2)
UIP-like pattern on HRCT, n/N (%)	96 (48.0)	25 (40.3)	15 (48.4)	14 (48.3)	10 (45.5)	6 (20.7)	166 (44.5%)

Data are n (% of patients with available data) or median (Q1, Q3)

Patients with missing data: *n* = 6 for ILD diagnosis; *n* = 1 for sex; *n* = 15 for race; *n* = 18 for history of smoking; *n* = 75 for time from ILD diagnosis to enrolment; *n* = 23 for family history of ILD; *n* = 23 for prior hospitalization; *n* = 34 for FEV_1_; *n* = 32 for FVC L; *n* = 52 for FVC % predicted; *n* = 61 for DLco mL/min/mmHg; *n* = 68 for DLco % predicted; and *n* = 118 for UIP-like pattern on HRCT

**Table 2:** Patient-reported outcomes at enrolment into the ILD-PRO Registry

	Autoimmune disease-associated ILDs (*n* = 229)	HP (*n* = 85)	iNSIP (*n* = 44)	IPAF (*n* = 43)	Unclassifiable ILDs (*n* = 37)	Other ILDs (*n* = 47)	Total (*n* = 491)
SF-12 physical component summary score	34.1 (25.7, 40.4)	34.4 (25.4, 39.2)	34.5 (28.6, 42.3)	37.3 (28.1, 41.0)	35.5 (28.4, 47.5)	30.7 (24.1, 34.9)	34.1 (26.1, 40.5)
SF-12 mental component summary score	49.4 (39.8, 57.2)	54.9 (46.0, 60.9)	46.8 (38.5, 58.2)	48.5 (42.9, 56.3)	54.8 (41.9, 59.8)	46.0 (37.4, 57.5)	50.1 (40.7, 57.7)
CASA-Q cough symptoms domain score	58.3 (41.7, 75.0)	58.3 (33.3, 79.2)	58.3 (33.3, 75.0)	58.3 (29.2, 75.0)	50.0 (41.7, 75.0)	50.0 (33.3, 83.3)	58.3 (33.3, 75.0)
CASA-Q cough impact domain score	68.8 (50.0, 90.6)	68.8 (43.8, 90.6)	68.8 (46.9, 93.8)	73.4 (42.2, 92.2)	75.0 (46.9, 93.8)	68.8 (40.6, 96.9)	68.8 (46.9, 93.8)
EuroQoL index score	0.7 (0.6, 0.8)	0.8 (0.6, 0.9)	0.7 (0.5, 0.9)	0.8 (0.6, 0.8)	0.8 (0.6, 0.8)	0.7 (0.6, 0.8)	0.7 (0.6, 0.8)
EuroQoL VAS	60.5 (50.0, 80.0)	70.0 (50.0, 76.0)	60.0 (40.0, 80.0)	70.0 (50.0, 80.0)	67.0 (48.0, 75.0)	67.5 (40.0, 80.0)	65.0 (50.0, 80.0)
SGRQ total score	50.0 (35.8, 63.0)	50.2 (36.7, 65.6)	57.8 (30.6, 67.4)	48.1 (39.9, 60.1)	46.8 (33.2, 58.2)	58.3 (38.7, 69.8)	50.8 (35.9, 64.7)
SGRQ symptoms score	51.0 (35.1, 66.2)	52.6 (37.3, 67.9)	53.9 (40.0, 67.5)	48.1 (29.1, 67.9)	57.2 (38.0, 62.9)	66.2 (40.6, 78.6)	53.1 (35.6, 68.5)
SGRQ activity score	72.8 (59.5, 85.8)	78.3 (53.6, 85.8)	79.4 (47.7, 85.8)	73.4 (60.4, 85.8)	61.1 (47.7, 73.7)	72.8 (66.2, 85.8)	72.8 (54.5, 85.8)
SGRQ impact score	33.6 (18.8, 51.3)	34.7 (18.7, 53.3)	38.6 (18.6, 57.3)	37.3 (23.1, 49.4)	34.1 (21.0, 52.5)	43.8 (22.5, 61.7)	35.6 (19.2, 53.4)

Data are median (Q1, Q3). Six patients had missing data on ILD diagnosis. Patients with missing data: *n* = 101 for SF-12 summary scores; *n* = 29 for CASA-Q cough symptoms domain score; *n* = 30 for CASA-Q cough impact domain score; *n* = 38 for EuroQoL index score and VAS; *n* = 52 for SGRQ total score; *n* = 37 for SGRQ symptoms score; *n* = 42 for SGRQ activity score; *n* = 40 for SGRQ impact score. Lower SF-12 scores and EuroQoL scores indicate worse HRQL; lower CASA-Q scores indicate worse cough; higher SGRQ scores indicate worse HRQL

CASA-Q, Cough and Sputum Assessment Questionnaire. SF-12, 12-item short-form survey. SGRQ, St George’s Respiratory Questionnaire. VAS, visual analogue scale

### Patient characteristics by type of ILD

**Fig. 5:**
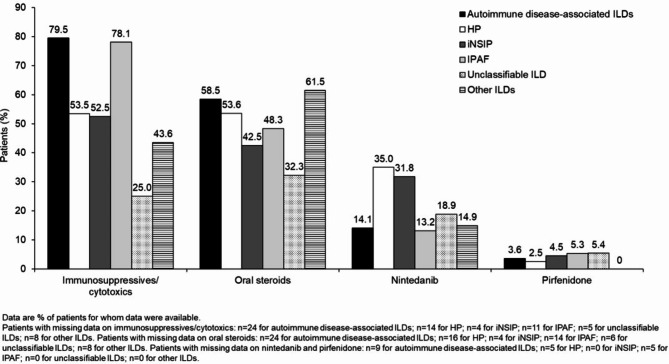
Use of immunosuppressives, oral steroids and antifibrotic therapies at enrolment into the ILD-PRO Registry by type of ILD

Among 485 patients with data on the type of ILD: 229 (47.2%) had autoimmune disease-associated ILDs, 85 (17.5%) HP, 44 (9.1%) iNSIP, 43 (8.9%) IPAF, 37 (7.6%) unclassifiable ILD and 47 (9.7%) other ILDs. The majority of patients with autoimmune disease-associated ILDs, HP, iNSIP and IPAF were female, whereas the majority of patients with unclassifiable ILD or other ILDs were male (Table 1). Median FVC % predicted across subgroups by type of ILD ranged from 56.7 to 68.6, while median DLco % predicted ranged from 37.1 to 42.8 (Table 1). Greater proportions of patients with autoimmune disease-associated ILDs (79.5%) and IPAF (78.1%) were receiving immunosuppressive/cytotoxic therapy than patients in the other subgroups (25.0–53.5%) (Fig. 5). Greater proportions of patients with HP (35.0%) and iNSIP (31.8%) were receiving nintedanib than patients in the other subgroups (13.2–18.9%) (Fig. 5). Few patients received pirfenidone (Fig. 5).

### Patient characteristics by time from ILD diagnosis to enrolment

Median time from ILD diagnosis to enrolment was 732 days. Compared with the subgroup with a shorter time (< 732 days) from ILD diagnosis to enrolment, the subgroup with a longer time (> 732 days) included a greater proportion of females, had a lower median FVC (L) and lower median DLco % predicted and included a greater proportion of patients with a relative decline in FVC % predicted ≥ 10% within the prior 24 months (Additional file 4). There were no significant differences in the proportions of patients with different types of ILD, the proportion with a history of smoking, or median FVC % predicted between patients with a shorter versus longer time from ILD diagnosis to enrolment. Patients with a longer versus shorter time from ILD diagnosis to enrolment were more likely to be receiving immunosuppressive or cytotoxic therapy (68.0% vs. 57.5%). There were numerical but non-significant differences in the proportions of patients with a longer versus shorter time from ILD diagnosis to enrolment who were taking oral steroids (57.1% vs. 47.0%, respectively) or nintedanib (24.4% vs. 19.3%, respectively).

### Comparison of patients in the ILD-PRO Registry and IPF-PRO Registry

Compared with the patients in the IPF-PRO Registry, a significantly greater proportion of patients in the ILD-PRO Registry were female, while smaller proportions had a history of smoking or were white (Additional file 5). Patients in the ILD-PRO Registry had lower FVC % predicted (62.2 vs. 69.8) and DLco % predicted (39.2 vs. 42.3) and worse HRQL (based on all the patient-reported outcomes assessed) at enrolment than patients in the IPF-PRO Registry, and were more likely to have hiatal hernia, pulmonary hypertension, asthma, heart failure and chronic kidney disease (Additional file 5). Greater proportions of patients in the ILD-PRO Registry than IPF-PRO Registry were receiving immunosuppressive or cytotoxic therapy (64.5% vs. 1.4%), while smaller proportions were receiving nintedanib (19.8% vs. 24.3%) or pirfenidone (3.6% vs. 30.3%).

## Discussion

The prospective multicentre ILD-PRO Registry will evaluate the course, management, and impact of PPF in clinical practice in the US. Data from the first 491 patients enrolled show that the participants have a variety of ILD diagnoses, marked impairment in lung function and HRQL, and high medication use. Almost two-thirds of the patients were receiving immunosuppressive or cytotoxic therapy at enrolment, reflecting the widespread off-label use of these therapies in patients with ILDs other than IPF, particularly in patients with autoimmune diseases. Use of nintedanib was substantially higher than use of pirfenidone, reflecting that only nintedanib has been approved in the US for the treatment of chronic fibrosing ILDs with a progressive phenotype other than IPF.

Clinical trials and observational studies have demonstrated progressive loss of lung function in patients with PPF [7, 8, 15–17], with variation in the rate of decline among subgroups and individuals [17–20]. Consistent with this, patients with a longer time from ILD diagnosis to enrolment into the ILD-PRO Registry had worse lung function and were more likely to have experienced significant loss of FVC within the prior 24 months than those with a shorter time from ILD diagnosis to enrolment. At enrolment, the patients in the ILD-PRO Registry had similar or worse lung function than the patients with PPF enrolled in clinical trials [4, 5, 21]. Longitudinal data from this registry will further our knowledge of the long-term course of PPF and predictors of more rapid progression.

Patient registries play a key role in improving understanding of rare diseases by providing long-term follow-up data from large and heterogeneous patient cohorts, including patients who would not have been eligible to participate in clinical trials [22]. The data collected in the ILD-PRO Registry will add to those generated by other registries involving patients with pulmonary fibrosis, including the German INSIGHTS-ILD and EXCITING-ILD registries [23, 24], the Canadian Registry for Pulmonary Fibrosis (CARE-PF) [25], the Pulmonary Fibrosis Foundation Patient Registry (PFF-PR) [26] and the Australasian ILD registry [27]. Data from these registries have already provided insights about the course of PPF. For example, data from the Pulmonary Fibrosis Foundation registry have shown that Black patients were diagnosed later and died at a younger age than Hispanic and White patients [28] and that greater impairment in quality of life due to cough is associated with higher risks of respiratory-related hospitalization and death [29]. Data from the CARE-PF registry have shown that exposure to particulate matter is associated with decline in FVC and mortality [30] and that workplace productivity loss correlates with the severity of symptoms [31]. Trajectories of FVC observed in the EXCITING-ILD registry showed that the course of ILD is heterogeneous and that lower FVC and higher age at baseline were associated with disease progression [32].

The ILD-PRO Registry is unique among registries in that it has specifically enrolled patients who meet criteria for PPF, rather than all patients with ILDs. Other notable features of this registry include a call centre, which will help minimise missing data, the storage of digital HRCT scans for qualitative and quantitative assessment, and the collection of blood samples. Analyses of bio-samples from the IPF-PRO Registry have identified circulating biomarkers associated with disease severity and progression in patients with IPF [33–35]. Similarly, analyses of bio-samples from the ILD-PRO Registry may help identify or validate circulating biomarkers that are predictive of poor outcomes in patients with different types of progressive fibrosing ILD.

Like all real-world studies, the ILD-PRO Registry has design elements that will need to be considered when evaluating the data that it provides. Different clinicians may use different methodologies and come to different decisions when making a differential diagnosis of ILD, particularly in the case of diagnoses such as iNSIP versus unclassifiable ILD. The evaluation and diagnosis of comorbidities such as gastroesophageal reflux disease will also differ among clinicians. Identification of PPF also presents challenges, given the timing of clinic visits, pulmonary function tests and HRCT scans in clinical practice. In patients enrolled into the ILD-PRO Registry, the criteria for PPF could have been met at any time within the prior 24 months, meaning that patients may have been enrolled some time after they developed PPF. Investigators were asked to select a single criterion for ILD progression that best applied to the subject, but in reality a patient may meet several criteria for progression. Patients are being enrolled into the ILD-PRO Registry mainly at specialist ILD centres and may not be representative, in terms of their clinical characteristics or management, of the general population of patients with PPF.

## Conclusion

The ILD-PRO Registry is a prospective multicentre US registry of patients with PPF. Longitudinal data from this registry will provide valuable insights into the course, impact and management of different forms of PPF.

A graphical abstract of the data presented in this manuscript is available in the Supplementary appendix (Additional file 1).

## Electronic supplementary material

Below is the link to the electronic supplementary material.

Supplementary Material 1: **Additional file 1**: Graphical abstract. **Additional file 2**: Enrolling centers and PIs. **Additional file 3**: Immunosuppressant or cytotoxic therapies received at enrollment into the ILD-PRO Registry. **Additional file 4**: Characteristics that differed significantly between patients with shorter vs longer times from ILD diagnosis to enrollment into the ILD-PRO Registry. **Additional file 5**: Characteristics that differed significantly between patients enrolled into the ILD-PRO Registry and IPF-PRO Registry.

## Data Availability

The datasets analysed during the current study are not publicly available, but are available from the corresponding author on reasonable request.

## Abbreviations

BIPI: Boehringer Ingelheim Pharmaceuticals, Inc
CASA-Q: Cough and Sputum Assessment Questionnaire
DCRI: Duke Clinical Research Institute
DLco: Diffusing capacity of the lungs for carbon monoxide
FVC: Forced vital capacity
HP: Hypersensitivity pneumonitis
HRCT: High-resolution computed tomography
ILD: Interstitial lung disease
iNSIP: Idiopathic non-specific interstitial pneumonia
IPAF: Interstitial pneumonia with autoimmune features
IPF: Idiopathic pulmonary fibrosis
PPF: Progressive pulmonary fibrosis
SF-12: 12-item short-form survey
SGRQ: St. George’s Respiratory Questionnaire
VAS: Visual analogue scale
