# Supplementary material for: Design and baseline characteristics of the ILD-PRO registry in patients with progressive pulmonary fibrosis
Source: BMC Pulm Med. 2024 Sep 27;24:468. doi: 10.1186/s12890-024-03247-8 (PMC11438290; doi:10.1186/s12890-024-03247-8)
Supplement: Supplementary file 1 — Supplementary Material 1: Additional file 1: Graphical abstract. Additional file 2: Enrolling centers and PIs. Additional file 3: Immunosuppressant or cytotoxic therapies received at enrollment into the ILD-PRO Registry. Additional file 4: Characteristics that differed significantly between patients with shorter vs longer times from ILD diagnosis to enrollment into the ILD-PRO Registry. Additional file 5: Characteristics that differed significantly between patients enrolled into the ILD-PRO Registry and IPF-PRO Registry. [file 12890_2024_3247_MOESM1_ESM.docx]

**Design and baseline characteristics of the ILD-PRO Registry in patients with progressive pulmonary fibrosis**

**Supplementary appendix**

**Additional file 1:** Graphical abstract.


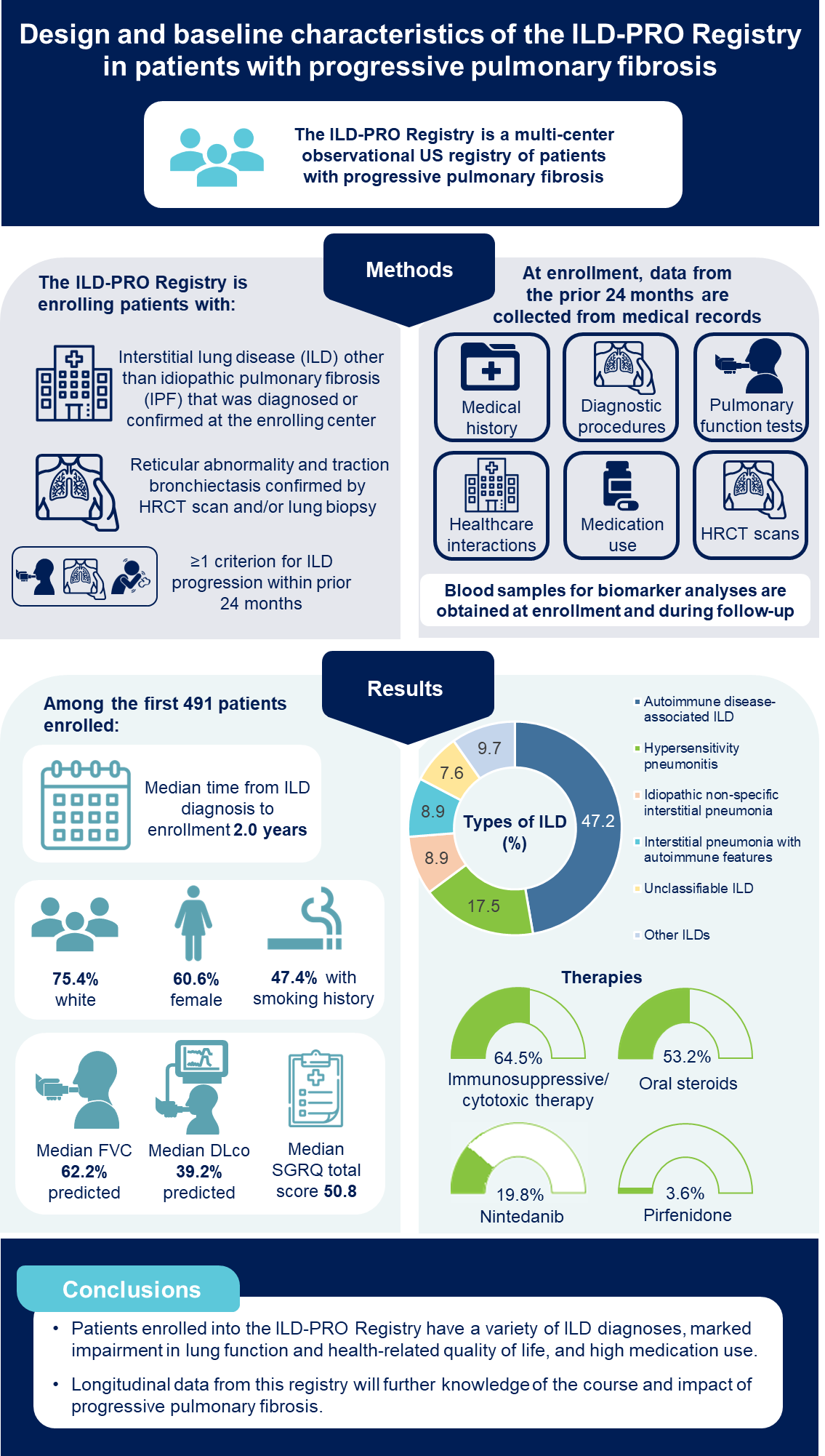


**Additional file 2:** Enrolling centres and PIs.

Albert Baker, Lynchburg Pulmonary Associates, Lynchburg, VA; Debabrata Bandyopadhyay, University of South Florida, Tampa, FL; Scott Beegle, Albany Medical Center, Albany, NY; John A Belperio, University of California Los Angeles, Los Angeles, CA; Domingo Chardon, Ponce Research Institute, Ponce, Puerto Rico; Rany Condos, NYU Medical Center, New York, NY; Daniel Dilling, Loyola University Health System, Maywood, IL; Jane Dematte, Northwestern University, Evanston, IL; John Fitzgerald, UT Southwestern Medical Center, Dallas, TX; Kevin R Flaherty, University of Michigan, Ann Arbor, MI; Reginald Fowler, Salem Chest and Southeastern Clinical Research Center, Winston-Salem, NC; Mridu Gulati, Yale School of Medicine, New Haven, CT; Nishant Gupta, University of Cincinnati Medical Center, Cincinnati, OH; Amy Hajari Case, Piedmont Healthcare, Austell, GA; Mark Hamblin, University of Kansas, Lawrence, KS; David Hotchkin, The Oregon Clinic, Portland, OR; Robert J Kaner, Weill Cornell Medical College, New York, NY; Jad Kebbe, University of Oklahoma, Norman, OK; Hyun J Kim, University of Minnesota, Minneapolis, MN; Marta Kokoszynska, University of Vermont Lung Center, Colchester, VT; Lisa H Lancaster, Vanderbilt University Medical Center, Nashville, TN; Joseph A Lasky, Tulane University, New Orleans, LA; Doug Lee, Wilmington Health and PMG Research, Wilmington, NC; Timothy Liesching, Lahey Clinic, Burlington, MA; Randolph Lipchik, Froedtert & The Medical College of Wisconsin Community Physicians, Milwaukee, WI; L Jason Lobo, UNC Chapel Hill, Chapel Hill, NC; Tracy R Luckhardt, University of Alabama at Birmingham, Birmingham, AL; Yolanda Mageto, Baylor University Medical Center at Dallas, Dallas, TX; Toby M Maher, University of Southern California, Los Angeles, CA; Lake Morrison, Duke University Medical Center, Durham, NC; Andrew Namen, Wake Forest University, Winston Salem, NC; Tessy Paul, University of Virginia, Charlottesville, VA; Mary Porteous, University of Pennsylvania, Philadelphia, PA; Rishi Raj, Stanford University, Stanford, CA; Murali Ramaswamy, PulmonIx LLC, Greensboro, NC; Jesse Roman, Thomas Jefferson University, Philadelphia, PA; Ivan Rosas, Baylor College of Medicine, Houston, TX; Tonya Russell, Washington University, St. Louis, MO; Zeenat Safdar, Houston Methodist Lung Center, Houston, TX; Namita Sood, University of California, Davis, Davis, CA; Brian Southern, Cleveland Clinic, Cleveland, OH; Mary E Strek, University of Chicago, Chicago, IL; Srihari Veeraraghavan, Emory University, Atlanta, GA; Rajat Walia, St. Joseph’s Hospital, Phoenix, AZ; Timothy PM Whelan, Medical University of South Carolina, Charleston, SC.

**Additional file 3:** Immunosuppressant or cytotoxic therapies received at enrolment into the ILD-PRO Registry.

|  | **n (%)** |
| --- | --- |
| Patients with data on type of immunosuppressive or cytotoxic therapy | 198 (100) |
| Mycophenolate mofetil | 101 (51.0) |
| Azathioprine | 35 (17.7) |
| Hydroxychloroquine | 28 (14.1) |
| Other | 34 (17.2) |

**Additional file 4** Characteristics that differed significantly between patients with shorter vs longer times from ILD diagnosis to enrolment into the ILD-PRO Registry.

|  | **Shorter time between ILD diagnosis and enrolment (<732 days) (n=208)** | **Longer time between ILD diagnosis and enrolment (>732 days) (n=208)** | **P-value** |
| --- | --- | --- | --- |
| **Sex** | | |  |
| Female | 106 (51.0) | 135 (65.2) | 0.003 |
| **Inclusion criteria re: ILD progression** |  |  |  |
| Relative decline in FVC % predicted ≥10% | 93 (44.7) | 121 (58.2) | <0.001 |
| Relative decline in DLco % predicted ≥10% | 39 (18.8) | 35 (16.8) |  |
| Relative decline in FVC % predicted ≥5–<10% plus worsened respiratory symptoms | 19 (9.1) | 28 (13.5) |  |
| Relative decline in FVC % predicted ≥5–<10% plus increased extent of fibrotic changes on HRCT | 4 (1.9) | 3 (1.4) |  |
| Worsened respiratory symptoms plus increased extent of fibrotic changes on HRCT | 53 (25.5) | 21 (10.1) |  |
| **Lung function** |  |  |  |
| FVC, L | 2.2 (1.7, 2.9) | 2.0 (1.6, 2.4) | <0.001 |
| FEV_1_, L | 1.8 (1.4, 2.3) | 1.5 (1.2, 2.0) | <0.001 |
| DLco, mL/min/mmHg | 10.8 (8.3, 14.5) | 9.9 (7.4, 12.4) | <0.001 |
| DLco, % predicted | 39.2 (30.5, 50.1) | 37.7 (29.3, 47.5) | 0.035 |
| **Patient-reported outcomes** | | |  |
| SGRQ activity score | 72.8 (51.0, 81.2) | 78.3 (60.0, 85.8) | 0.047 |
| **Comorbidities** | | |  |
| Hiatal hernia | 31 (15.3) | 52 (26.4) | 0.006 |
| Deep vein thrombosis or pulmonary embolism | 6 (3.0) | 17 (8.6) | 0.015 |
| **Medications** | | |  |
| Immunosuppressive/cytotoxic drugs | 104 (57.5) | 119 (68.0) | 0.038 |

Data are n (% of patients with available data) or median (Q1, Q3). Characteristics for which p<0.05 for difference between groups with shorter and longer times from ILD diagnosis to enrolment are shown. Patients with missing data in shorter time subgroup: n=10 for FVC and FEV_1_; n=22 for DLco mL/min/mmHg; n=26 for DLco % predicted; n=15 for SGRQ activity score; n=5 for comorbidities; n=27 for immunosuppressive/cytotoxic drugs. Patients with missing data in longer time subgroup: n=1 for sex; n=15 for FVC; n=17 for FEV_1_; n=26 for DLco mL/min/mmHg; n=28 for DLco % predicted; n=19 for SGRQ activity score; n=11 for comorbidities; n=33 for immunosuppressive/cytotoxic drugs. St George’s Respiratory Questionnaire.

**Additional file 5** Characteristics that differed significantly between patients enrolled into the ILD-PRO Registry and IPF-PRO Registry.

|  | **ILD-PRO Registry**  **(n=491)** | **IPF-PRO Registry (n=1002)** | **P-value** |
| --- | --- | --- | --- |
| **Demographics** | | |  |
| Female | 297 (60.6) | 254 (25.3) | <0.001 |
| Race |  |  |  |
| White | 359 (75.4) | 930 (94.2) | <0.001 |
| Black/African-American | 89 (18.7) | 18 (1.8) |  |
| Other | 28 (5.9) | 39 (4.0) |  |
| Smoking history | 224 (47.4) | 670 (66.9) | <0.001 |
| **Lung function** | | |  |
| FVC, L | 2.0 (1.6, 2.7) | 2.7 (2.2, 3.3) | <0.001 |
| FVC, % predicted | 62.2 (49.4, 72.4) | 69.8 (59.7, 80.2) | <0.001 |
| FEV_1_, L | 1.7 (1.3, 2.1) | 2.2 (1.8, 2.7) | <0.001 |
| DLco, mL/min/mmHg | 10.3 (7.8, 13.4) | 12.2 (9.4, 15.3) | <0.001 |
| DLco, % predicted | 39.2 (30.2, 49.2) | 42.3 (32.6, 51.2) | 0.021 |
| **Patient-reported outcomes** | | |  |
| SF-12 physical component summary | 34.1 (26.1, 40.5) | 38.9 (31.2, 46.0) | <0.001 |
| SF-12 mental component summary | 50.1 (40.7, 57.7) | 53.7 (45.7, 59.2) | <0.001 |
| CASA-Q cough symptoms domain | 58.3 (33.3, 75.0) | 58.3 (41.7, 75.0) | 0.008 |
| CASA-Q cough impact domain | 68.8 (46.9, 93.8) | 78.1 (56.3, 93.8) | <0.001 |
| EuroQoL index score | 0.7 (0.6, 0.8) | 0.8 (0.7, 1.0) | <0.001 |
| EuroQoL VAS | 65 (50, 80) | 75 (60, 85) | <0.001 |
| SGRQ total score | 50.8 (35.9, 64.7) | 39.6 (25.5, 53.7) | <0.001 |
| SGRQ symptoms score | 53.1 (35.6, 68.5) | 43.2 (30.2, 61.0) | <0.001 |
| SGRQ activity score | 72.8 (54.5, 85.8) | 59.5 (41.4, 72.8) | <0.001 |
| SGRQ impact score | 35.6 (19.2, 53.4) | 26.1 (14.2, 42.1) | <0.001 |
| **Comorbidities** | | |  |
| Coronary artery disease | 101 (21.4) | 297 (29.8) | <0.001 |
| Hiatal hernia | 102 (21.6) | 168 (16.9) | 0.036 |
| Pulmonary hypertension | 85 (18.0) | 71 (7.1) | <0.001 |
| Asthma | 79 (16.8) | 74 (7.4) | <0.001 |
| Heart failure | 48 (10.1) | 58 (5.8) | 0.006 |
| Chronic kidney disease | 36 (7.6) | 32 (3.2) | 0.001 |
| **Medications** | | |  |
| PPIs | 278 (61.1) | 554 (55.5) | 0.044 |
| Statins | 212 (46.6) | 564 (56.6) | <0.001 |
| Bronchodilators | 222 (48.8) | 317 (31.9) | <0.001 |
| Oral steroids | 222 (53.2) | 129 (13.0) | <0.001 |
| Nintedanib | 93 (19.8) | 243 (24.3) | 0.049 |
| Pirfenidone | 17 (3.6) | 304 (30.3) | <0.001 |
| Immunosuppressive/cytotoxic drugs | 273 (64.5) | 14 (1.4) | <0.001 |
| Anticoagulants | 73 (16.0) | 210 (21.1) | 0.018 |
| H2 blockers | 68 (14.9) | 107 (10.7) | 0.029 |

Data are n (% of patients with available data) or median (Q1, Q3). Characteristics for which p<0.05 for difference between patients in the ILD-PRO Registry and IPF-PRO Registry are shown. Patients with missing data in ILD-PRO Registry: n=1 for sex; n=15 for race; n=18 for smoking history, coronary artery disease, hiatal hernia, pulmonary hypertension, heart failure, chronic kidney disease; n=32 for FVC, L; n=52 for FVC % predicted, SGRQ total score; n=34 for FEV_1_; n=61 for DLco mL/min/mmHg; n=68 for DLco % predicted, immunosuppressive/cytotoxic drugs; n=101 for SF-12; n=29 for CASA-Q cough symptoms; n=30 CASA-Q cough impact; n=38 for EuroQoL; n=37 for SGRQ symptoms score; n=42 for SGRQ activity score; n=40 for SGRQ impact score; n=20 for asthma; n=36 for PPIs, statins, bronchodilators, anticoagulants, H2 blockers; n=74 for oral steroids; n=21 for nintedanib, pirfenidone. Patients with missing data in IPF-PRO Registry: n=15 for race; n=1 for smoking history, nintedanib; n=59 for FVC, L, FEV_1_; n=73 for FVC % predicted; n=104 for DLco mL/min/mmHg; n=105 for DLco % predicted; n=92 for SF-12; n=48 for CASA-Q cough symptoms; n=49 CASA-Q cough impact; n=55 for EuroQoL index, SGRQ activity score; n=59 for EurQoL VAS; n=76 for SGRQ total score; n=60 for SGRQ symptoms score; n=57 for SGRQ impact score; n=4 for coronary artery disease, PPI, H2 blockers; n=7 for hiatal hernia, heart failure; n=6 for pulmonary hypertension, asthma; n=5 for chronic kidney disease, statins; n=8 for bronchodilators, oral steroids, anticoagulants; n=10 for immunosuppressive/cytotoxic drugs. CASA-Q, Cough and Sputum Assessment Questionnaire. PPI, proton pump inhibitors. SF-12, 12-item short-form survey. SGRQ, St George’s Respiratory Questionnaire. VAS, visual analogue scale.
